# Albany Medical College

**Published:** 1870-05

**Authors:** 


### *Albany Medical College.*

Dr. THOS. C. DURANT of New York, a graduate of the College and an early student of Drs. March and Armsby, has given $15,000 to endow the "*March Professorship.*"

Drs. E. R. Peaslee, and Meredith Clymer, of New York, and Dr. Wm. P. Seymour, of Troy, have accepted Chairs in the Faculty of the College.

